# Successful implantation of a Micra leadless pacemaker via collateral femoral vein and inferior vena cava filter

**DOI:** 10.1002/ccr3.1386

**Published:** 2018-01-30

**Authors:** Erica Flores, Mayur Patel, Geoffery Orme, Wilber Su

**Affiliations:** ^1^ Department of Cardiology University of Arizona College of Medicine – Phoenix 1111 E. McDowell Road Phoenix 85006 Arizona; ^2^ Department of Internal Medicine University of Arizona College of Medicine – Phoenix 1111 E. McDowell Road Phoenix 85006 Arizona

**Keywords:** Atrial fibrillation, complete heart block, inferior vena cava filter, pacemaker, tortuous venous supply, vascular occlusion

## Abstract

This case details the successful implementation of a leadless pacemaker device in a patient with multiple venous occlusions and an IVC filter. As the incidence of IVC filters increases in patients with dysrhythmias, further investigations are required to determine the risk and safety of leadless pacemaker placement in this population.

## Introduction

Managing dysrhythmias in complicated vascular patients can pose a unique challenge. The utilization of traditional transvenous pacemaker leads is associated with short‐ and long‐term complications, particularly central venous obstruction, which can reduce vascular access 1, 2. Epicardial lead placement eliminates venous access; however, this procedure requires minimally invasive surgery, exposing patients to peri‐operative risks, general anesthesia, and long‐term complications of lead exit block 3.

The Medtronic Micra transcatheter pacemaker system (TPS; Medtronic, Minneapolis, MN) is a leadless single‐chamber pacemaker that appears to be a suitable alternative option, especially when traditional approaches have failed. Although the implementation of this device has shown to be effective with high success and low complication rates, the study design only utilized a transfemoral venous approach and excluded patients with inferior vena cava filters 4, 5. As a result, complex vascular cases such as this patient with bilateral subclavian vein, superior vena cava, and bilateral common femoral vein occlusions make delivery of this novel device difficult. This report describes the first Micra leadless pacemaker implantation via a tortuous collateral branch of right common femoral vein requiring serial dilation and passage through previously placed inferior vena cava filter.

## Case Report

The patient is a 40‐year‐old male with past medical history significant for osteogenesis imperfecta, end‐stage renal failure with failed renal transplant on peritoneal dialysis, protein C/S deficiency, and recurrent pulmonary emboli with a Greenfield IVC filter placement in 2005. His medications included warfarin, nifedipine, pantoprazole, gabapentin, cinacalcet, calcitriol, calcium carbonate, calcium citrate, and ergocalciferol. Prior history included atrial fibrillation and complete heart block, and the patient had previously undergone failed endocardial pacing systems bilaterally, followed by an epicardial lead placement in 2015 with revisions due to a high threshold and early battery drain. His prior endocardial leads were extracted due to sepsis and infection. He then presented with episodes of loss of capture even at the highest pacing output after multiple refusals from surgeons to perform further lead revisions. Device check showed an underlying junctional escape rhythm at 30 beats per minute (bpm). The patient had previous AV fistulas placed in the upper and lower extremities, and imaging studies revealed chronic occlusions of bilateral subclavian, superior vena cava, and internal/external jugular veins. These chronic occlusions allowed for extensive collateral formation, and as a result, the patient remained asymptomatic with respect to his venous obstructions. In preparation for this procedure, the case was discussed with vascular surgery in the event that a complication arose.

**Figure 1:**
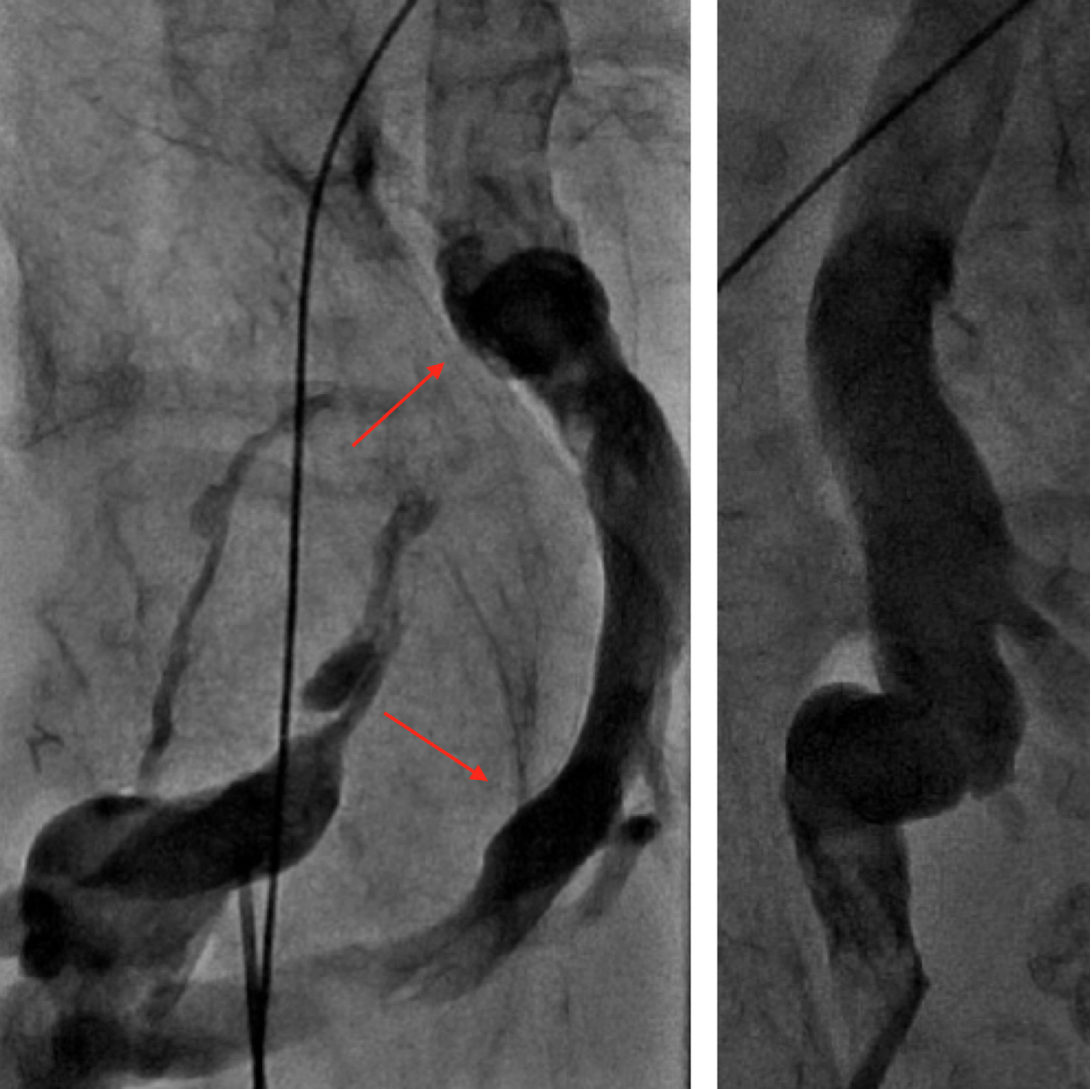
A venogram revealing an occluded right common femoral vein with a large deep collateral vein (arrows, left). A detailed fluoroscopy of the S‐shaped bend of the collateral vein (right).

**Figure 2:**
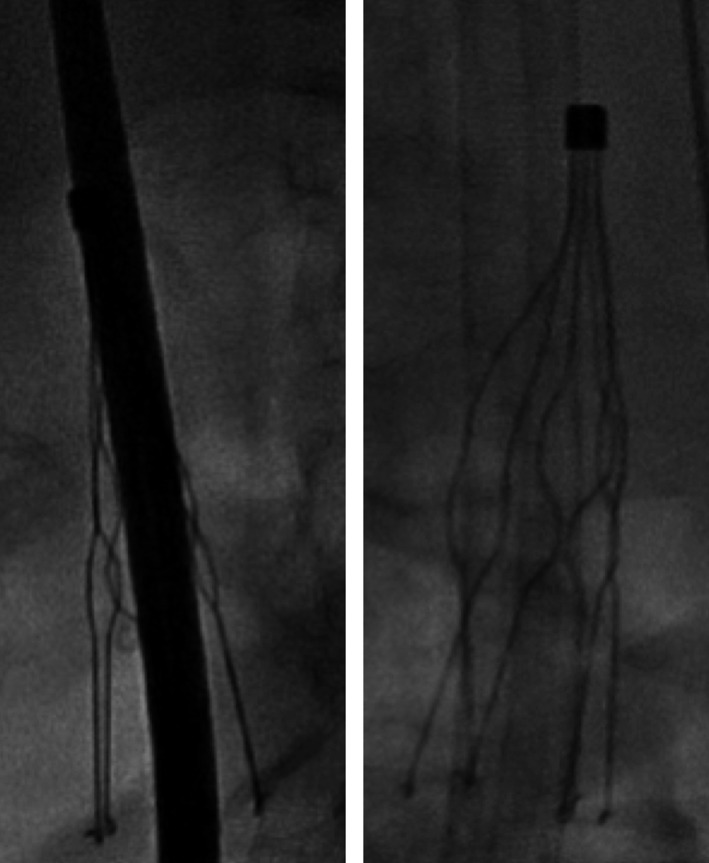
A 16‐French sheath (left) and 27‐French sheath (right) passing through Greenfield IVC filter.

**Figure 3:**
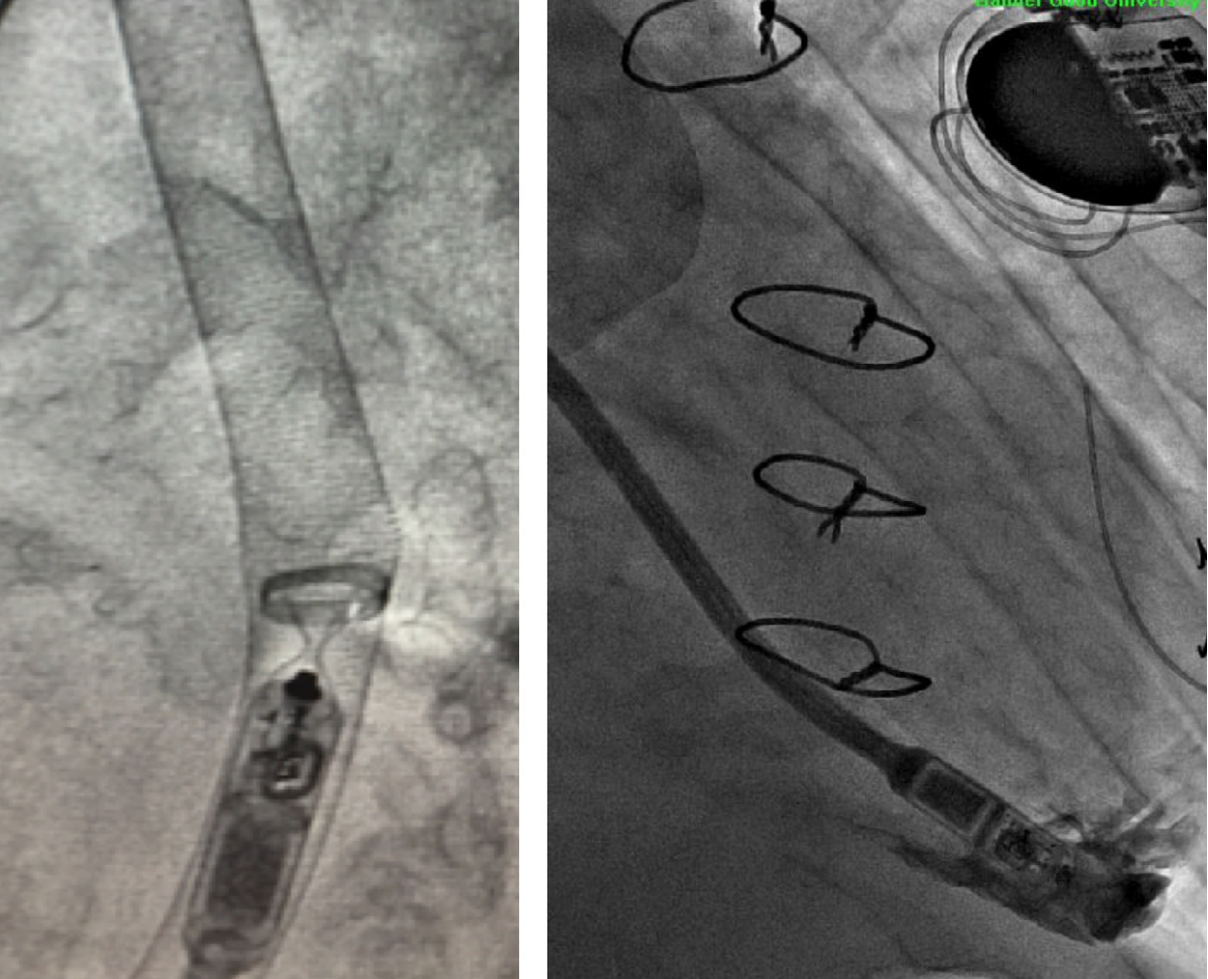
Migration of Micra TPS device through a kink in the 27‐French sheath (left) and deployment of the device in right ventricular apex (right).

Despite having an IVC filter in place, the patient's sole remaining pacing option was a transfemoral leadless pacemaker implantation. Preoperative laboratories demonstrated that patient had a supratherapeutic INR of 4.7; he was subsequently given vitamin K and his warfarin was held resulting in an INR of 1.9 on the day of the procedure. The delivery of this system became imperative when the patient's epicardial lead lost capture at the beginning of this procedure. The patient remained asymptomatic, but was now in a junctional rhythm at a rate of 20–30 beats per minute throughout the intervention. The right common femoral vein (CFV) was accessed under ultrasound guidance, and a venogram demonstrated an occluded right CFV with a large deep collateral that exhibited an acute S‐shaped bend causing the vein to course in different planes (Fig. 1). The left CFV venogram revealed a completely occluded vein and small collaterals. Thus, the collateral branch of the right CFV was the only identifiable access site. The narrowest portion of the venous collateral was 8.4 mm. On fluoroscopy, the angulation of the S‐shaped bend was approximately 70–80°; a flexible guidewire was used to maneuver through the curves. With the guidewire in place, a 16F dilator was advanced with difficulty, as there was resistance at each bend. Prior to traversing the IVC filter, additional imaging was obtained and the guidewire was exchanged to an Amplatz super stiff wire. The 16F dilator was then passed between the legs of the filter. Serial dilations with gradually larger sheaths were made to accommodate the 27F introducer sheath, which passed between the same legs of the filter (Fig. 2). The Medtronic Micra™ pacemaker device was successfully deployed into the right ventricle apex with adequate capture (Fig. 3). After removal of the sheath, there was no displacement of the IVC filter or the individual legs under fluoroscopy. The patient's pacemaker generator was removed after the placement of the Micra device, and epicardial leads were capped. There were no bleeding complications postprocedure, and his warfarin was resumed the following day. The capture threshold for the Micra device the day after implantation and at 1‐year follow‐up was 0.38 V at 0.24 msec.

## Discussion

Considerable options for permanent pacemaker placement in this patient included a femoral transvenous lead placement, epicardial lead revision, or implantation of Micra leadless pacemaker. After implementation of a transvenous pacemaker, the incidence of venous obstruction is approximately 12–15% 2, 6. This patient's risk was undoubtedly higher given his thrombophilia due to protein C/S deficiency. There is literature describing venous occlusions in patients with osteogenesis imperfecta; however, thrombosis is not a commonly associated cardiovascular feature related to this disease. Thus, placing a long femoral vein lead through an IVC filter carried an undue risk as a nidus to further propagate thrombus burden. The success rate of a third epicardial lead revision was low for this patient after weighing the potential complications of the procedure and the high pacing threshold required due to ventricular scarring 3. The Micra device, which is the only Federal Drug Administration approved leadless pacemaker in the USA, also has the potential for thrombus formation. However, we believed it carried a relatively lower thrombotic risk and provided the only long‐term pacing solution compared to the alternative options. Therefore, the Micra leadless pacemaker implantation was utilized.

The first challenge with this approach was obtaining appropriate vascular access as the bilateral common femoral veins were occluded. Due to the utilization of such large diameter sheaths, the common femoral venous approach has been the only studied approach for the delivery of the Micra TPS device with vascular complication rates of 0.7% 7. Selecting an alternative venous access was inconceivable in this patient as his upper extremity was inaccessible due to chronic thrombus formation. Despite this fact, gaining access to any upper extremity venous supply with the known advancement of a 27F introducer sheath would have led to unpredictable complications. Therefore, the decision was made to pursue the collateral branch of the right common femoral vein. The difficulty with this collateral vein was the tortuosity of the vessel, which has impaired previous Micra TPS implantation attempts 7. As the introducer sheath is a relatively rigid structure, particularly at the apex, any significant tortuosity increases the risk of developing kinks in the sheath. These kinks can displace or fracture the flexible nitinol tines and impede the overall delivery of the Micra TPS device to the right ventricle. Therefore, our goal was to serially dilate the vessel in order to minimize the amount and the degree of curvature present prior to the insertion of the 27F introducer sheath. Despite our best efforts, numerous kinks in the introducer sheath occurred (Fig. 3). Our solution to mitigate this dilemma involved pulling back the sheath while simultaneously advancing the Micra TPS device at every kink. This case demonstrated the challenges encountered with advancing a 27F introducer sheath and Micra TPS device through a tortuous venous supply. The risk of vascular complications, particularly perforation and bleeding, is certainly higher in this scenario than previously described. However, the unique circumstances surrounding this case justified the additional risk, as the implementation of the Micra TPS was the sole option for this patient. In the future, when the Micra device reaches EOS, surgical revision will be explored; however, given his complex vascular anatomy, his most plausible option would be a second Micra device implantation.

Furthermore, the presence of an IVC filter is a relative contraindication in the implantation of the Micra leadless pacemaker as it utilizes a transfemoral venous approach. This reasoning is due to the risk of filter dislodgement, guidewire entrapment, and perforation. Yet, there are multiple case reports dating from 1991 to 2013 that have utilized a transfemoral venous approach and crossed an IVC filter in order to complete a multitude of complex interventional cardiac procedures 8. A compilation of all case reports during this period included 48 patients, of which 29 had Greenfield IVC filters 8. These historical data suggest that crossing an IVC filter safely is possible. However, the upper limit of sheath size deemed safe to pass through a filter has not been described in the literature. This is the second case to report advancement of a 27F sheath across an IVC filter. Of note, the filter design in our case (Greenfield; Boston Scientific, Natick, MA) differs compared to the first (Bird's Nest filter; Cook Medical, Bloomington, IN) 9, 10. The Bird's Nest filter is constructed of four preshaped and nonmatching stainless steel wires that are fixed at each end to V‐shaped struts 11. In contrast, the Greenfield Filter is constructed of six zigzag‐shaped legs that are fitted to an apical hub and positioned in a radial array 12. The base of this filter is 3 cm in diameter, and the legs are separated by 11 mm in the deployed position 12. Thus, a 27F sheath (9 mm diameter) can theoretically pass between the legs of the Greenfield Filter, which we have demonstrated. Based on this experience, a 27F is likely the upper limit of sheath size that can safely pass through Greenfield IVC filters. It is unclear if the risk of the Micra TPS device implantation alters with IVC filter design. As with the first case, there was no migration or fracture of the IVC filter during the procedure and there was no noted compromise in filter function after 1‐year follow‐up. As the incidence of IVC filters increases in patients with dysrhythmias, further investigations are required to determine the risk and safety of this procedure in any IVC filter design.

## Conclusion

This is the first reported case of a Medtronic Micra™ pacemaker device successfully implemented via a collateral branch of the right common femoral vein along with previously placed Greenfield IVC filter. We have shown that transfemoral deployment of such a large pacemaker device in a patient with a complex vascular anatomy and a different designed IVC filter is possible, but must be undertaken with caution.

## Authorship

EF, MP, GO, and WS: helped draft the manuscript and then revised the manuscript, and gave final approval for the manuscript to be published and take full responsibility for the content of the paper.

## Conflict of Interest

Dr. Wilber Su receives research grants and honorarium from Medtronic.

## References

[1] Udo, E. O. , N. P. Zuithoff , N. M. van Hemel , C. C. de Cock , T. Hendriks , P. A. Doevendans , et al. 2012 Incidence and predictors of short‐ and long‐term complications in pacemaker therapy: the FOLLOWPACE study. Heart Rhythm 9:728–735. 22182495 10.1016/j.hrthm.2011.12.014

[2] Bracke, F. , A. Meijer , and B. Van Gelder . 2003 Venous Occlusion of the access vein in patients referred for lead extractions: Influence of patient and lead characteristics. Pacing Clin. Electrophysiol. 26:1649–1652. 12877695 10.1046/j.1460-9592.2003.t01-1-00247.x

[3] Auricchio, A. , C. Stellbrink , S. Sack , M. Block , J. Vogt , P. Bakker , et al. 2002 Long‐term clinical effect of hemodynamically optimized cardiac resynchronization therapy in patients with heart failure and ventricular conduction delay. J. Am. Coll. Cardiol. 39:2026–2033. 12084604 10.1016/s0735-1097(02)01895-8

[4] El‐Chami, M. F. , P. R. Roberts , A. Kypta , P. Omdahl , M. D. Bonner , R. C. Kowal , et al. 2006 How to Implant a Leadless Pacemaker With a Tine‐Based Fixation. J. Cardiovasc. Electrophysiol. 27:1495–1501. 10.1111/jce.13092 27600684

[5] El‐Chami, M. , R. C. Kowal , K. Soejima , P. Ritter , G. Z. Duray , P. Neuzil , et al. 2017 Impact of operator experience and training strategy on procedural outcomes with leadless pacing: insights from the Micra Transcatheter Pacing Study. Pacing Clin. Electrophysiol. 40:834–842. 28439940 10.1111/pace.13094

[6] Spittell, P. C. , and D. L. Hayes . 1992 Venous complications after insertion of a transvenous pacemaker. Mayo Clin. Proc. 67:258–265. 1545594 10.1016/s0025-6196(12)60103-7

[7] Reynolds, D. , G. Z. Duray , R. Omar , K. Soejima , P. Neuzil , S. Zhang , et al. 2016 A leadless intracardiac transcatheter pacing system. N. Engl. J. Med. 374:533–541. 26551877 10.1056/NEJMoa1511643

[8] Jez, J. , Z. Starek , F. Lehar , J. Wolf , and M. Novak . 2015 Complex electrophysiology intervention in a patient with inferior vena cava filter. Cor Vasa 57:341–346.

[9] Afzal, M. R. , J. Ackers , J. D. Hummel , and R. Augostini . 2017 Safety of implantation of a leadless pacemaker via femoral approach in the presence of an inferior vena cava filter. Pacing Clin. Electrophysiol. 40:975–976. 28213895 10.1111/pace.13052

[10] Grewal, S. , M. R. Chamarthy , and S. P. Kalva . 2016 Complications of inferior vena cava filters. Cardiovasc. Diagn. Ther. 6:632–641. 28123983 10.21037/cdt.2016.09.08 PMC5220210

[11] Reed, R. A. , G. P. Teitelbaum , F. C. Taylor , M. J. Pentecost , and J. O. Roehm . 1991 Use of the Bird's Nest filter in oversized inferior venae cavae. J. Vasc. Interv. Radiol. 2:447–450. 1797210 10.1016/s1051-0443(91)72216-1

[12] Greenfield, L. J. , M. C. Proctor , and K. R. Roberts . 1997 An improved process for development and testing of vena caval filters: the percutaneous steel Greenfield filter. Surgery 121:50–57. 9001551 10.1016/s0039-6060(97)90182-3

